# Both IL-1*β* and TNF-*α* Regulate NGAL Expression in Polymorphonuclear Granulocytes of Chronic Hemodialysis Patients

**DOI:** 10.1155/2010/613937

**Published:** 2011-03-03

**Authors:** Adriana Arena, Giovanna Stassi, Daniela Iannello, Domenica Gazzara, Maria Calapai, Carlo Bisignano, Davide Bolignano, Antonio Lacquaniti, Michele Buemi

**Affiliations:** ^1^Unit of Clinical Microbiology, Department of Surgical Science, Faculty of Medicine, University of Messina, 98125 Messina, Italy; ^2^Pharmaco-Biological Department, University of Messina, 98125 Messina, Italy; ^3^Department of Internal Medicine, University of Messina, 98125 Messina, Italy

## Abstract

*Background*. NGAL is involved in modulation of the inflammatory response and is found in the sera of uremic patients. We investigated whether hemodiafiltration (HDF) could influence the ability of polymorphonuclear granulocytes (PMGs) to release NGAL. The involvement of interleukin- (IL-)1*β* and tumor necrosis factor- (TNF-)*α* on NGAL release was evaluated. *Methods*. We studied end-stage renal disease (ESRD) patients at the start of dialysis (Pre-HDF) and at the end of treatment (Post-HDF) and 18 healthy subjects (HSs). Peripheral venous blood was taken from HDF patients at the start of dialysis and at the end of treatment. *Results*. PMGs obtained from ESRD patients were hyporesponsive to LPS treatment, with respect to PMG from HS. IL-1*β* and TNF-*α* produced by PMG from post-HDF patients were higher than those obtained by PMG from pre-HDF. Neutralization of IL-1*β*, but not of TNF-*α*, determined a clear-cut production of NGAL in PMG from healthy donors. On the contrary, specific induction of NGAL in PMG from uremic patients was dependent on the presence in supernatants of IL-1*β* and TNF-*α*. *Conclusion*. Our data demonstrate that in PMG from healthy subjects, NGAL production was supported solely by IL-1*β*, whereas in PMG from HDF patients, NGAL production was supported by IL-1*β*, TNF-*α*.

## 1. Introduction

Neutrophil gelatinase associated lipocalin (NGAL) is a 25-kDa glycoprotein first found, as a matrix protein, in specific granules of human neutrophils [1]. NGAL is involved in a variety of cellular processes, including the innate immune response, [2–4]. NGAL expression is also found in epithelial cells, where it is strongly induced in the presence of inflammation [5, 6]. Furthermore, the protein has been associated with several tumor types, including breast, ovarian, colorectal, and pancreatic cancer [7–10].

NGAL expression is rapidly induced in the nephron in response to renal epithelial injury [11, 12], it was recently reported that NGAL levels are predictive of [13, 14] the onset of acute renal injury following treatments that are potentially harmful to the kidney and also the serious exacerbation of unstable nephropathy. Furthermore, recently reported findings also suggest that NGAL may be involved in the pathophysiological process underpinning chronic renal conditions such as polycystic kidney disease [15] and glomerulonephritis [16]. NGAL levels, clearly correlated with the severity of renal impairment, probably express the degree of active damage underlying the chronic condition. Among the several concepts concerning the role of inflammation in acute kidney injury that have recently emerged are alterations in the endothelial and epithelial renal cells of the kidney as consequence of proinflammatory mediators. The interaction between innate and adaptive immunity contributes to the inflammation, thus leading to renal parenchymal cell death and acute renal injury. In the early steps of this process, a key role is played by neutrophils, which are important mediators of innate immunity. Since polymorphonuclear granulocytes (PMGs) are the predominant infiltrating cell type present in the acute inflammatory response, they act as a first line of defence against invading microorganisms, as well as in the inflammation status, associated with the renal endothelial damage occurring in patients chronic kidney disease. Moreover, it has been demonstrated that NGAL exerts its antimicrobial effect by binding iron-transporting molecules, thus it could play an important role in antimicrobial defence in uremic patients [17]. The mediators orchestrating the inflammatory response are cytokine networks, the main controlling elements in the immune reactions. Among these cytokines, interleukin (IL-)1*β*  and tumor necrosis factor- (TNF-)*α* have profound effects on the proinflammatory process peculiar to acute kidney injury.

Previous reports also suggested that NGAL is upregulated in response to inflammation. In particular, Cowland et al. have demonstrated that NGAL is selectively upregulated in human epithelial cells by the IL-1*β* but not by TNF-*α* in an NF-*κ*B-dependent manner [18]. 

Furthermore, other Authors have demonstrated that IL-1*β* plasma levels are increased in long-term *hemodiafiltration *patients [19], and several groups have also found increased circulating TNF-*α* levels in patients undergoing hemodiafiltration [20], although others have not made this finding [21]. 

The main aim of the present study was therefore to investigate whether intermittent HDF in end-stage renal disease patients (ERDS) can influence the release of NGAL by polymorphonuclear granulocytes (PMGs) obtained from pre and postHDF patients. A further end-point was to evaluate IL-1*β* and TNF-*α* production, and evaluate any role they might play in NGAL modulation.

 In this *in vitro* study we present, for the first time, evidence that the specific induction of this innate immune defence protein, in HDF patients, depends mainly on the presence of Il-1*β* and TNF-*α*  in PMG supernatants *.*

## 2. Materials and Methods

Thirty chronic HDF patients were enrolled in the study; all had been dry-weight stable for at least 2 months before the study was started and had achieved a normotensive edema-free state. Exclusion criteria were: presence, or a recent history, of bleeding, malignancy, liver, thyroid or infectious diseases, alterations in the leukocyte count or formula and/or recent treatment with steroids or immunosuppressors. 

Patients had been treated with hemodiafiltration (HDF) with the same prescription for 6 months: three times a week for 3.5–4 hours, blood flow 300 mL/min; bicarbonate infusion 2000 mL/h; mean weight loss set at 2.5 kg. HDF was conducted using the Integra Machine (Hospal, Bologna, Italy). 

 Peripheral venous blood was taken from HDF patients at the start of dialysis (PreHDF) and at the end of treatment (Post-HDF) and from a small group of 18 healthy subjects (HSs) matched with HDF patients for age and gender. The study was approved by the local Ethics Committee, and fully informed consent was obtained from all participants.

### 2.1. Isolation of Human Polymorphonuclear Granulocytes (PMGs)

PMG were isolated from freshly collected venous blood. PMGss were isolated from peripheral blood anticoagulated in Heparin, using Mono-Poly Resolving Medium (M-PRM) following the manufacturer's instructions (MP Biomedical, Illkirch, France). Briefly, M-PRM is a solution composed of a polysaccharide (Ficoll 400) and a radiopaque contrast medium (Hypaque) in a specific ratio to yield a density of 1.114 + 0.002. Blood was centrifuged at 300× g for 30 minutes. After centrifugation, the following fractions were obtained: mononuclear leucocyte band, PMG band and the the red blood cell pellet. The PMG band was harvested and washed three times in RPMI 1640 medium, was cultured in 24-well plates at a concentration of 2 × 10^6^ cells/mL per well in RPMI 1640 medium supplemented with 50 *μ*g/mL gentamicin and 5% fetal calf serum (FCS), at 37°C in a 5% CO_2_ atmosphere. All reagents were supplied by Sigma Aldrich (Milan, Italy).

### 2.2. Treatments

Lipopolysaccharide (LPS) from the E.coli strain 055:B5 was used as the positive control. LPS was used at a concentration of 1 *μ*g/mL in or not in the presence of recombinant proteins or monoclonal antibodies.

 18, 24, and 48 hours post treatment, the supernatants were harvested, and suitable aliquots were stored at −80°C until cytokine analysis.

### 2.3. Cytotoxicity Test

To determine cells viability 18 h, 24 h, and 48 h after culture a colorimetric assay was used as described by Mosmann [22]. The assay is based on the tetrazolium salt 3-(4,5 dimethylthiazol-2-yl) 2,5diphenyltetrazoliumbromide (MTT), a pale yellow substrate that is cleaved by active mitochondria to produce a dark blue formazan product. Briefly, cells were seeded onto 96-well culture plates at a number of 10^4^ per well. The plate was then incubated at 37°C in an atmosphere of 5% CO_2_ for 18 h, 24 h, and 48 h. The medium was then discarded and the MTT reagent added. The plate was reincubated at 37°C for an additional 3 h to allow formazan development. The plates were read with a microelisa reader using a wavelength of 570 nm. The percentage of cytotoxicity was calculated as follows:

(1) $$1-\;\left[\frac{\left(\text{experiment}\;\text{OD}-\text{lysis}\;\text{control}\;\text{OD}\right)}{\left(\text{cell}\;\text{control}\;\text{OD}-\text{lysis}\;\text{control}\;\text{OD}\right)}\right]\times 100.$$

### 2.4. Limulus Test

Culture media and reagents tested for the presence of endotoxin using the E-Toxate kit (Sigma, Milan) were found to contain <10 pg of endotoxin per mL.

### 2.5. Cytokine Evaluations

Supernatants from PMG in different experimental conditions, were harvested, centrifuged and kept at −80°C until titration for the presence of TNF-*α* and IL-1*β* by an immunoenzymatic method (ELISA); the kits used were supplied by R&D System (Milan, Italy) and NGAL (BioPorto Diagnostics, Verona, Italy), respectively. The minimum detectable dose of TNF-*α* was less than 1.6 pg/mL, of IL-1*β* less than 1 pg/mL, and NGAL, less than 1 pg/mL.

### 2.6. Cytokines and Monoclonal Antibodies

The concentrations used were 1 ng/mL for recombinant human (rh)IL-1*β* and 10 ng/mL for recombinant human (rh)TNF-*α.*

 Monoclonal antihuman TNF-*α* (mAbvsTNF-*α*) (ND50 was 0.015–0.06 *μ*g/mL in the presence of 0.25 ng/mL of recombinant human TNF-*α*) and monoclonal antihuman IL-1*β*  (mAbvsIL-1*β*) (the ND50 for this antihuman IL-1*β* antibody was determined to be approximately 0.05–0.1 *μ*g/mL in the presence of 50 pg/mL of rhIL-1*β* on using the D10.G4.1 cell proliferation assay) were added to human PMG at the time of LPS treatment. All reagents were supplied by R&D System (Milan, Italy). 

 The concentration of antibody required to neutralize IL-1*β* and TNF-*α* activity depended on the cytokine concentration obtained.

### 2.7. Statistical Evaluation

Results are expressed as the means of three experiments ± standard deviation (S.D.). Data were analysed using one-way analysis of variance (ANOVA) and the Student-Newman-Keuls test. Differences were considered statistically significant at a *P* value of <.05.

## 3. Results

The main characteristics of the study cohort patients are summarized in Table 1. 

Table 2 shows the kinetics (18, 24, and 48 hours) of IL-1*β* and TNF-*α* release by PMG from different donors. No basal production of IL-1*β* and TNF-*α* was found in any of the groups examined. LPS triggered PMG from different donor groups to release markedly high levels of IL-1*β* and TNF-*α*. In particular, the levels of both cytokines in supernatants of PMG from HS were significantly higher than those from pre and postHDF (*P* < .05). Furthermore, the levels of IL-1*β* and TNF-*α* from postHDF PMG were higher than those obtained by PMG from preHD (*P* < .05). The kinetics of IL-1*β* and TNF-*α* showed a production peak at 24 hours post LPS-stimulation in all the experimental conditions. Incubation times (18, 24, and 48 hours) did not significantly influence cell viability (data not shown). 

**Figure 1:**
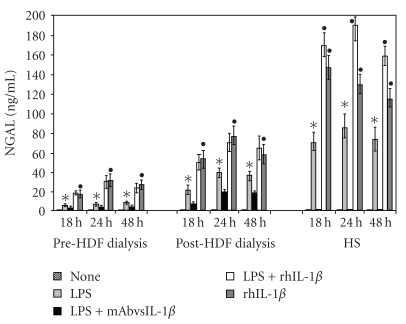
Role of IL-1*β* on the kinetics of NGAL production by PMG from preHDF and postHDF patients and HS. *Significantly different (*P* < .05) from that of unstimulated PMG. °Significantly different (*P* < .05) from that of LPS-stimulated PMG. ^•^Significantly different (*P* < .05) from that of LPS-stimulated PMG.

Figure 1 reports the results concerning the role of IL-1*β* on NGAL production. No basal production of NGAL was found in PMG from preHDF and postHDF patients or HS.

LPS-stimulation of PMG induced a significant upregulation in NGAL, both in uremic patients and in HS with respect to unstimulated PMG (*P* < .05). When recombinant IL1*β*  were added to unstimulated PMG, an upregulation of NGAL production was obtained in all groups with respect to that obtained with LPS treatment (*P* < .05). Moreover, the addition of rhIL-1*β* to PMG LPS-stimulated induced levels of NGAL similar to those obtained in PMG treated with rhIL-1*β* in pre and postdialysis patients, whereas in PMG from HS combined treatment with LPS and rhIL-1*β* determined a greater production of NGAL than that in patients treated solely with rhIL-1*β* (*P* < .05). 

In the attempt, prompted by the above findings, to gain further insight into the role of IL-1*β* on NGAL modulation it was found that the neutralization of IL-1*β*  by monoclonal antibodies in LPS-stimulated PMG determining a 50% decrease of NGAL production in predialysis patients (*P* < .05), and a 60% decrease in postdialysis patients (*P* < .05). Whereas, the neutralization of IL-1*β* determined a clearcut production in PMG from healthy subjects with respect to LPS treated PMG (*P* < .05). Is interesting to address that in all the experimental conditions, PMG from preHDF patients produced lower amounts of NGAL compared with those from postHDF patients; levels were even lower with respect to PMG from HS. The NGAL kinetics showed a peak in production at 24 hours in all the experimental conditions. 

In the light of the above data, we investigated whether the amounts of TNF-*α* found in supernatants of PMG from all the groups studied (Table 1) might be involved in modulating NGAL production. 

**Figure 2:**
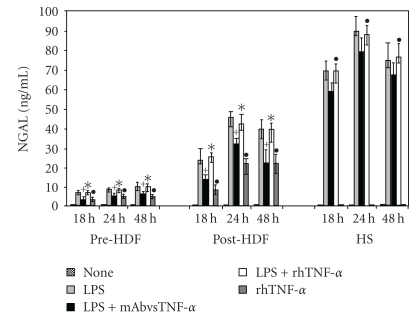
Role of TNF-*α* on the kinetics of NGAL production by PMG from preHDF and postHDF patients and HS. ^+^Significantly different (*P* < .05) from that of LPS-stimulated PMG. *Significantly different (*P* < .05) from that of rhTNF-alpha-treated PMG. °Significantly different (*P* < .05) from that of unstimulated PMG. ^•^Significantly different (*P* < .05) from that of rhTNF-alpha-treated PMG.

The data reported in Figure 2 show the TNF-*α*  effect on NGAL release by PMG from different donor groups. 

The addition of rhTNF-*α* to unstimulated PMG determined an upregulation of NGAL production only in PMG from pre and postHDF (*P* < .05). On the contrary, in cells from HS the addition of rhTNF-*α* failed to trigger the production of NGAL.

Moreover, the addition of rhTNF-*α*  to LPS-stimulated PMG induced higher levels of NGAL in pre and postHDF compared to that observed in PMG treated with rhTNF*α* alone (*P* < .05). In PMG from healthy donors, combined treatment (LPS/rhTNF-*α*) determined the appearance of a marked amount of NGAL with respect to amounts observed after rhTNF-*α* treatment (*P* < .05). 

Furthermore, the neutralization of TNF-*α* by monoclonal antibodies in LPSstimulated PMG determined a down-regulation of NGAL production in cells from pre and post dialysis patients (*P* < .05). On the contrary, in PMG from HS the neutralization of TNF-*α* did not have any effect on NGAL production. The kinetics of NGAL showed a peak in production at 24 hours in all the experimental conditions.

**Figure 3:**
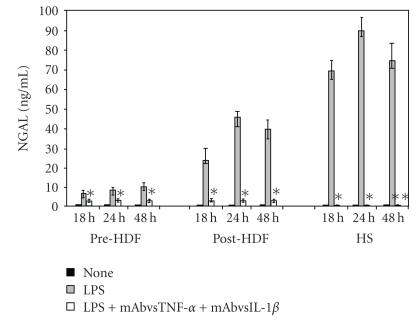
Effects of neutralization of IL-1*β* and TNF-*α* on the kinetics of NGAL production by PMG from preHDF and postHDF patients and HS. *Significantly different (*P* < .05) from that of LPS-stimulated PMG. **Significantly different (*P* < .05) from that of LPS-stimulated PMG.

In order to confirm the hypothesis that NGAL release in PMG from HDF patients was supported by IL-1*β* and TNF-*α*, in another series of experiments, we neutralized both cytokines. The results obtained are shown in Figure 3. Unexpectedly, after the neutralization of IL-1*β* and TNF-*α*, the PMG from uremic patients still produced appreciable amounts of NGAL, albeit smaller than the amounts induced by LPS (*P* < .05). On the contrary, the neutralization of IL-1*β* and TNF-*α* in PMG from healthy donors determined a clearcut production of NGAL (*P* < .05).

## 4. Discussion

In addition to having metabolic and endocrinal functions, renal tubule cells appear to probably play an important role in the systemic inflammatory balance, participating in the complex and dynamic network of leukocyte action and pro and antiinflammatory cytokines. Loss of this function may result in a propensity to develop systemic inflammatory response syndrome, and may relate to chronic inflammatory state in end-stage renal disease [23, 24].

 It is well known that complement, TLRs, numerous cytokines and chemokines are clearly involved in amplifying the immune response to kidney injury [25, 26]. A body of evidence in literature indicates that both innate and adaptive immunity are involved in uremic patients. The innate immune system, is activated very early in infectious or inflammatory states in a non-antigen-specific fashion, and is comprised of neutrophils, monocytes/macrophages, dendritic cells (DC) and natural killer (NK) cells. In contrast, the adaptive immune system, which becomes responsive to specific antigens over the course of several days, and includes DC, T and B lymphocytes. It is well known that the interaction between the different immune cells is mediated by a complex network of cytokines and chemokines with pleiotropic effects that orchestrate the immune response [27]. Thus, the induction, perpetuation, and collapse of a particular cytokine network and of the cellular events that it controls are strongly influenced by the dynamic relationships between pro and antiinflammatory cytokines, as well as their rates of production [28]. 

Neutrophils may play an early and critical role in patients with uremia. In fact, numerous cytokines may induce the synthesis of a variety of other inflammatory mediators, most of which are chemotactic for neutrophils,, which are then recruited to and activated in the inflammatory focus, this may contribute in inducing tissue damage. Among the factors, involved in this process, a key role appears to be played by an innate immune defence protein, NGAL. 

In the present paper, we evaluated whether HDF in end-stage renal disease patients can influence *in vitro* cultured PMG in producing NGAL. In addition, we analyzed the role of IL-1*β* and TNF-*α* in order to ascertain whether they are involved in NGAL production. The data reported demonstrate that PMG obtained from ESRD patients on hemodialysis are hyporesponsive to LPS treatment; their impaired immune response is characterized by the lower IL-1*β* and TNF-*α* with respect to PMG production in healthy subject. These findings seem to be in disagreement with data reported by Cowland et al. [18]. that demonstrate that LPS was not able to induce NGAL expression. However, this different behaviour could be ascribed to the different cellular system used. In fact, those experiments were carried out on epithelial cell lines and ours on PMG obtained from peripheral blood. Furthermore, our results demonstrate that the levels of IL-1*β* and TNF-*α* produced by PMG from postHDF patients were higher than those obtained by PMG from preHDF. We believe that this depends on hemodialysis restoring the impaired immune PMG status. In fact, PMG collected from patients at the end of the dialysis session displayed a markedly greater capacity to respond *in vitro* to LPS-stimulation than PMG collected during the preHDF session. 

Interestingly, in all the experimental conditions, the amounts of NGAL produced by LPS-stimulated PMG from preHDF patients were lower than those produced by PMG from postHDF patients and much smaller than those produced by PMG from HS. Furthermore, no significant differences were found between the kinetics of NGAL production in LPS-stimulated PMG obtained from preHDF patients at the different assay times. On the contrary, in LPS-stimulated PMG obtained from postHDF patients and HS, peak in the kinetics of NGAL production occurred at 24 hours following the trend of IL-1*β* and TNF-*α* production. These findings prompted us to further investigate the role of IL-1*β* and TNF-*α* in NGAL modulation.

Our findings demonstrate that the addition of rhIL-1*β* to PMG upregulates NGAL production in both uremic patients and HS whereas, the addition of rhTNF-*α* to PMG can increase NGAL production only in uremic patients. These data demonstrate that NGAL production by PMG from HS is supported solely by the presence of IL-1*β*  in cell supernatants. On the other hand, in PMG from uremic patients, NGAL production appeared to be supported mainly by IL-1*β* and TNF-*α* too. To verify this, we conducted another series of experiments, neutralizing both IL-1*β* and TNF-*α* with monoclonal antibodies. The neutralization of IL-1*β* and TNF-*α* in PMG from HS determined a clearcut production of NGAL. Unexpectedly, however, the neutralization of IL-1*β* and TNF-*α* did not completely eliminate NGAL production in PMG from HDF-patients; in fact, appreciable amounts of NGAL were still produced.

Overall, our findings demonstrate that in PMG from HS, NGAL production is supported solely by IL-1*β*, whereas in PMG from HDF patients, NGAL production is supported by IL-1*β*, TNF-*α*  and also by other biological mediator(s).

Recently, Karlsen et al. [29] demonstrated that NGAL was strongly induced by stimulation with TNF-*α* in the presence of IL-17, a proinflammatory cytokine. We can hypothesize that results concerning the effect of TNF-*α* on NGAL production by PMG from uremic patients could be related to a dynamic relationship between Th-1 and Th-17 response. Further studies are needed in order to understand this intriguing network of biological mediators and the consequent cellular events that they control. 

The open question is the role that other biological mediators may play in PMG from uremic patients in determining the nature of cytokine networks and thus in determining the quality and quantity of microenvironmental signals involved in NGAL release.

## Figures and Tables

**Table 1:** Main characteristics of the study cohort.

Parameter	HDF patients (*n* : 30)	HSS (*n* : 18)
Gender (M/F)	16/14	10/8
Age (yrs)	55 ± 12	56 ± 9
Dialysis vintage (mos)	38 [8–299]	—
spKt/V (weekly mean)	1.21 ± 0.19	—
PCR (g/Kg/day)	1.18 ± 0.23	—
PTH (pg/mL)	188 [42–348]	—
Creatinine (mg/dL)	9.9 ± 2.1	0.9 ± 0.2
Urea (mg/dL)	177.4 ± 39.6	18.5 ± 3.3
Ca X P product (mg2/dL2)	46.6 ± 13.3	30.1 ± 1.9
Hemoglobin (g/dL)	11.6 ± 1.8	15.0 ± 2.0
Hematocrit (%)	31.9 ± 3.0	44.3 ± 3.7
Erythrocytes (*n* × 10^6^)	3.59 ± 0.98	4.93 ± 0.81
White Cells (*n* × 10^6^)	6.5 ± 1.6	7.8 ± 1.1
Albumin (g/dL)	4.22 ± 0.65	4.06 ± 0.43
hsCRP (mg/L)	6 [1–42]	0.15 [0.07–0.44]
*β*2-microglobulin (mg/dL)	29 [7–53]	0.12 ± 0.4
Uric acid (mg/dL)	6.02 ± 1.08	5.03 ± 0.77
Serum iron (mcg/mL)	59.9 ± 19.8	88.5 ± 18.1
Serum transferrin (mg/dL)	187.1 ± 45.0	300.9 ± 37.1
Serum ferritin (ng/mL)	155 [9–789]	151 ± 33

**Table 2:** Kinetics of IL-1*β* (pg/mL) and TNF-*α* (pg/mL) release by PMG from preHDF and postHDF patients and HS.

	IL-1*β* (pg/ml)			TNF-*α* (pg/ml)		
	18 h	24 h	48 h	18 h	24 h	48 h
preHDF						
none	<1	<1	<1	<1.6	<1.6	<1.6
LPS	70.1 ± 8.3	120.2 ± 15.4	32.7 ± 7.9	85.4 ± 15.3	123.7 ± 21.9	51.6 ± 20.9

postHDF						
none	<1	<1	<1	<1.6	<1.6	<1.6
LPS	407.5 ± 37.4**	511.8 ± 68.6**	331.3 ± 21.7**	286.2 ± 87.1**	481.4 ± 98.3**	192.5 ± 61.8**

HS						
none	<1	<1	<1	<1.6	<1.6	<1.6
LPS	797.5 ± 65.8*	810.7 ± 58.5*	550.3 ± 39.8*	1409.3 ± 99.1*	1847.3 ± 121.7*	1055.4 ± 111.6*

*Significantly different (*P* < .05) compared with those obtained from pre and postHD.

**Significantly different (*P* < .05) compared with those obtained from preHD.
